# Computed tomography-guided percutaneous transthoracic needle biopsy for solitary pulmonary nodules in diameter less than 20 mm

**DOI:** 10.1097/MD.0000000000010154

**Published:** 2018-04-06

**Authors:** Chunhua Xu, Qi Yuan, Chuanzhen Chi, Qian Zhang, Yuchao Wang, Wei Wang, Like Yu, Ping Zhan, Yong Lin

**Affiliations:** a Endoscopic Center of Nanjing Chest Hospital; b Clinical Center of Nanjing Respiratory Diseases and Imaging; c Department of Respiratory Medicine, Nanjing Jiangning Hospital, Nanjing, Jiangsu, China.

**Keywords:** CT-guided, diagnosis, percutaneous lung biopsy, solitary pulmonary nodules

## Abstract

To evaluate the diagnostic value of computed tomography (CT)-guided percutaneous lung biopsy for solitary pulmonary nodules (SPN) < 20 mm.

A total of 248 patients who were diagnosed a SPN of < 20 mm underwent CT-guided percutaneous transthoracic needle biopsy were reviewed.

Specimens of 248 patients were obtained successfully. Around 174 cases were proved to be malignancies and 74 cases of benign lesions by biopsy. About 178 malignancies (71.8%) and 70 benign lesions were proved by surgery and clinical course. The diagnostic accuracy was 96.8%. The diagnostic accuracy of large nodules group (>10 and < 20 mm) was 99.3%, higher than 93.5% of small nodules group (≤10 mm) with statistical significance. The incidence of phenmothorax and hemorrhage was 16.1% and 6.8%, respectively. No death-related complications happened. The incidence of phenmothorax was related to puncture times (*P = *.013) and the length of puncture needle in lung tissues (*P = *.019).

CT-guided percutaneous lung biopsy for SPN of < 20 mm is an efficient and safe diagnostic method.

## 1 Introduction

With the improvement of healthy awareness of the population and availability of imaging examinations, solitary pulmonary nodules (SPN) are increasingly detected with the widespread use of chest computed tomography scans. The prevalence of SPN ranges from 8% to 51% in heavy or long-term smoker aged 50 years or older as reported.^[1]^ An estimated 150,000 US cases of SPN are diagnosed each year.^[2]^ Determining whether they are malignant or benign is a difficult problem in current study of clinicians. An SPN is defined as a single, spherical or oval, well-circumscribed, radiographic opacity that measures ≤3 cm in diameter and is surrounded completely by aerated lung, and there are no associated atelectasis, pulmonary hilar enlargement, or pleural effusion.^[3,4]^

SPN was divided into benign and malignant nodules according to characteristics. Approximately 11.9% of nodules 11 to 20 mm in diameter and 29.7% of nodules with a diameter of 21 to 30 mm are malignant, which are usually bronchogenic carcinoma.^[5]^ The prognosis of lung cancer patients who were diagnosed at an advanced stage of disease was poor with a 5-year survival rate of only 15.6%, whereas the value rise to 80% in early stage patients undergone surgery comparatively.^[6]^ Therefore, rapid identification and resection of malignant SPN is crucial to increase the survival and improve the prognosis.

CT-guided percutaneous transthoracic needle biopsy (CT-PTNB) is an efficient and safe diagnostic method for SPN.^[7]^At present, researches about diagnostic value of CT-PTNB for nodules that measure < 20 mm in diameter still lack, for smaller size of lung nodule presents a high technical difficulty. To evaluate the diagnostic value of CT-PTNB for SPN of < 20 mm, we conducted 248 SPN patients and analysed the associated complications, the diagnostic value, and safety of CT-PTNB in the present study.

## 2 Materials and methods

### 2.1 Patients

**Figure 1:**
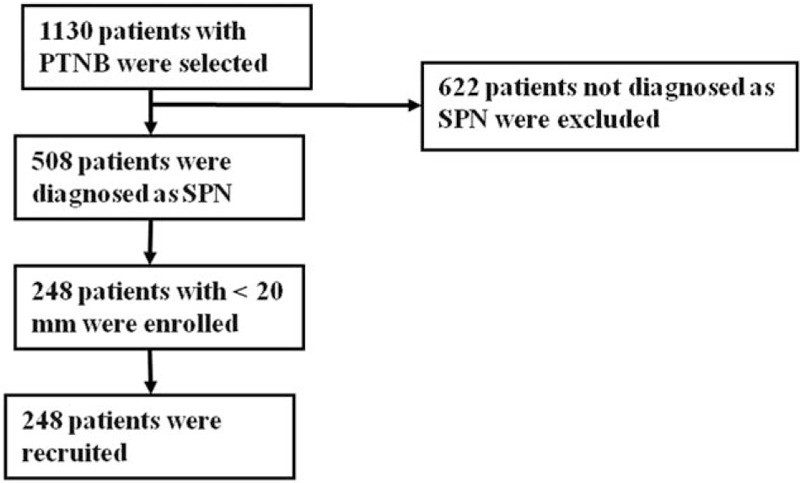
Flow diagram of the eligible patients and the interventional process of the study.

The study was designed to prospective evaluate the role of CT-PTNB for SPN of < 20 mm. Between January 2013 and January 2016, 1130 patients underwent percutaneous CT-guided lung biopsy at Endoscopic Center of Nanjing Chest Hospital. Of these, 248 consecutive cases identified as SPN of < 20 mm were enrolled. Inclusive criteria: SPN patients with the diameters measured < 20 mm using lung window settings, who have not been definitely diagnosed through bronchoscopy and CT scan. Exclusion criteria: Systemic immune disease, severe cardiopulmonary, renal or hepatic dysfunction, active hemorrhage and unable to finish the operation or any other medical or cognitive impairment that in the opinion of the investigator would interfere with the conduct of the study. All patients had undergone diagnostic chest CT in our hospital, before the biopsy (Fig. 1).

The study was approved by the Medical Ethics Committee of Nanjing Chest Hospital, Nanjing, China. All patients provided written informed consent before enrollment.

### 2.2 Procedure

All patients went through bleeding time and clotting time, blood routine, electrocardiogram before the procedure. The location of the lesions and adjacency relation with nearby vessels and organs were reviewed by contrast enhancement before CT-PTNB. All patients were trained breath and practiced breath-holding appropriately, and the cough one took antitussives preoperatively.

**Figure 2:**
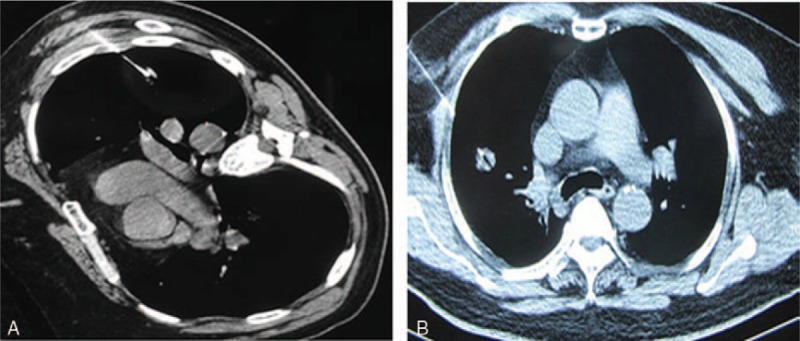
(A) CT-PTNB of the SPN lesion equal to 10 mm. (B) CT-PTNB of the SPN lesion equal to 18 mm. CT-PTNB = CT-guided percutaneous transthoracic needle biopsy, SPN = solitary pulmonary nodules.

Equipment used for CT-PTNB was 32-slice spiral CT (Aquilion, Toshiba, Tokyo, Japan), aspiration biopsy needle and tubular automatic biopsy gun (Cook, Bloomington, IN). Needle size was depending on the subjective visual assessment of the size and quality of the specimen. The patients underwent CT preoperatively in the prone, supine, or lateral position based on the shortest distance from the lesion to the body surface. The puncture entry point was marked on the skin through needle without penetrating the pleura assessed by CT scanning. The needle path length, chest wall thickness, needle-pleural angle was defined as the CT image showing. Direct the biopsy needle path away from sternum, rib and scapula, as well as large vessels and trachea. Needle was inserted in predetermined after local anaesthetic of 2% lidocaine was applied. Recheck the image when the needle close to the pleura, and then the biopsy procedure was continued immediately direction toward the lung lesion after adjusting the deviation and the pleura was penetrated. Fine needle aspiration and biopsy would be performed when thin-section CT scanning image of adjacent level showing the needle within the target lesion (Fig. 2). This procedure would typically performed twice occasionally in terms of tolerance of the patient and amount of resected specimen. Samples obtained by needle were smeared on a microscope glass slide and sent to the pathology laboratory after plunged into 4% formaldehyde solution to be fixed.

After removal of the biopsy needle, completion images were obtained to detect any post-biopsy complication. The patients were requested to avoid cough vigorously and stay supine and were only allowed to get off the bed 24 hours later. For patients developed a little pneumothorax post operation, chest x-ray was checked to observe pneumothorax absorption after bed rest and oxygen therapy in 1 week; Patients were ordered to stay in bed and take hemostatics if needle-path hemorrhage or small quantity of hemoptysis was found. The placement of a chest tube and anti-infection was considered in the event a patient became symptomatic and dyspnea, chest distress, massive hemorrhage, or a large (>30%) pneumothorax was found.

### 2.3 Diagnostic criteria

Malignant nodules: Patients whose biopsy histopathological findings showed malignancy and who received surgical resection were considered as malignant. Patients whose biopsy histopathological findings showed malignancy and with no surgical indication after systemic evaluation or with less willing to accept surgery were considered to be positive when the tumor size was reduced after radiotherapy, chemotherapy, or targeted therapy. Benign nodules: For patients whose biopsy histopathological findings showed a benign lesion, a true benign nature was demonstrated if the SPN had shrunk, disappeared, or remained unchanged after at least 1 year follow-up after suitable remedy.

### 2.4 Statistical analysis

Statistical analyses of the data were performed using SPSS version 21 (SPSS Inc., Chicago, IL). Measurement data were expressed by mean ± standard deviation. The diagnostic accuracy and complications rate were statistically compared for each influencing factor using bivariate logistic regression analyses and the Chi-square test.

Value of *P* < .05 was considered statistically significant.

## 3 Result

### 3.1 Clinical characteristics

A total of 248 patients with SPN were eligible for inclusion in this study (144 males and 104 females, mean age 58.5 years, ranged 35–76 years). The mean diameter of the lesion was 15 ± 4.8 mm. A total of 108 lesions (43.5%) were ≤10 mm and 140 lesions (56.5%) were of 10 to 20 mm in mean diameter. The localization of the SPN was the right upper lobe in 84 (33.9%), right middle lobe in 10 (4.0%), right lower lobe in 40 (16.1%), left upper lobe in 64 (25.8%), and left lower lobe in 50 (20.2%, Table 1).

**Table 1:**
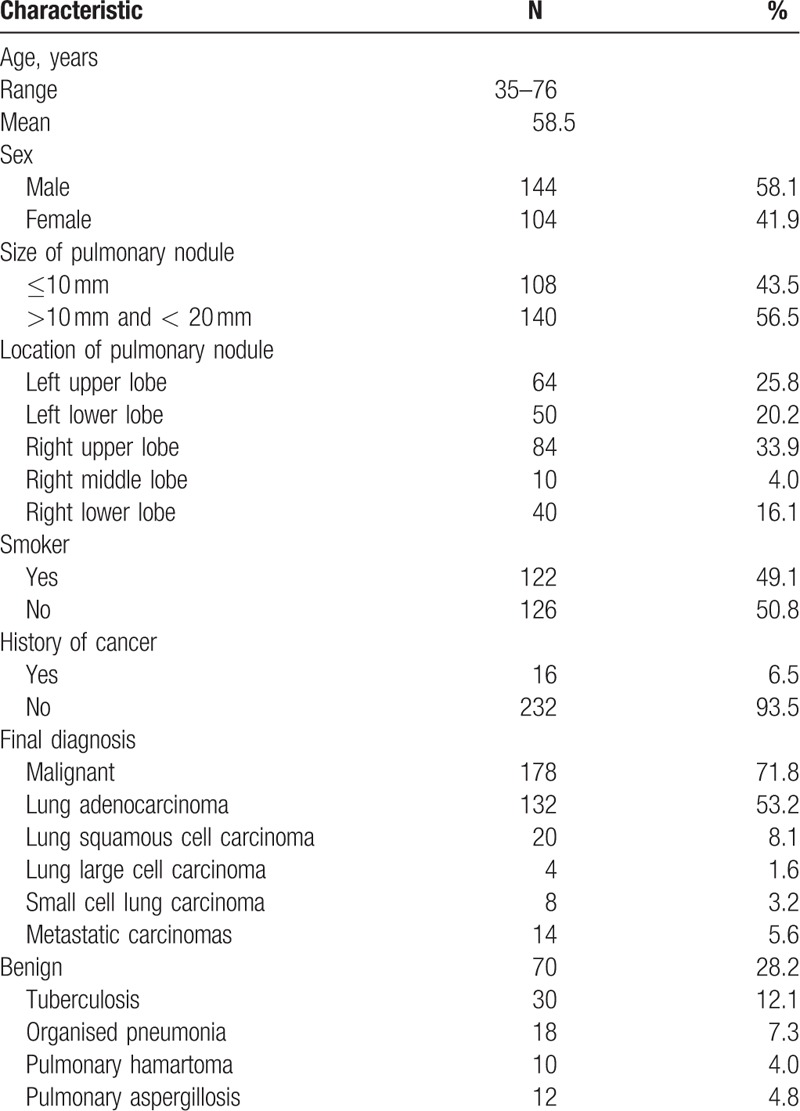
Patient demographics and nodule characteristics.

### 3.2 Diagnostic accuracy

Around 248 SPN lesions were punctured 283 times, of which 213 cases performed for once and respectively, 35 cases repeated for twice for the biopsy samples were not enough preliminarily. The final tissue diagnoses obtained for the total 248 patients were malignant lesion in 174, benign lesion in 74. By surgery or follow-up, false-positive cases occurred in 4/174 among the patients whose biopsy histopathological findings showed malignant, with 4 lung squamous cell carcinoma diagnosed by biopsy proved to be organised pneumonia. About 102 patients underwent surgical resection successfully, and postoperative pathology proved to be malignant, 68 patients who failed or unwilling to accept resection proved to be malignant when the reaction was assessed after radiotherapy, chemotherapy, or targeted therapy. False-negative cases occurred in 4/74 among the patients whose biopsy histopathological findings showed benign, with 1 pneumonia and 2 organised pneumonia diagnosed by biopsy proved to be lung adenocarcinoma after surgery, and 1 chronic inflammation revised to be lung squamous cell carcinoma after surgery. The final diagnoses and follow-up of the rest of the 70 benign patients were established as follows: 42 granuloma and 30 caseous necrosis shrinked to same extent after antituberculosis treatment. Around 12 pulmonary aspergillosis obviously dwindled after antifungal therapy and 18 organised pneumonia and 10 pulmonary hamartoma got smaller after anti-infection treatment or was unchanged in 1-year follow-up (Table 1). Around 178 cases (71.8%) of malignancies and 70 (28.2%) of benign lesions were proved by surgery and clinical course. The diagnostic accuracy was 96.8%. A total of 248 cases of SPN were divided into small nodule group (≤10 mm, n = 108) and the large nodules group (>10 mm and <20 mm, n = 140) according to nodule size. The diagnostic accuracy of large nodules group was 99.3%, higher than 93.5% of small nodules group with statistical significance (*P = *.023, Table 2).

**Table 2:**
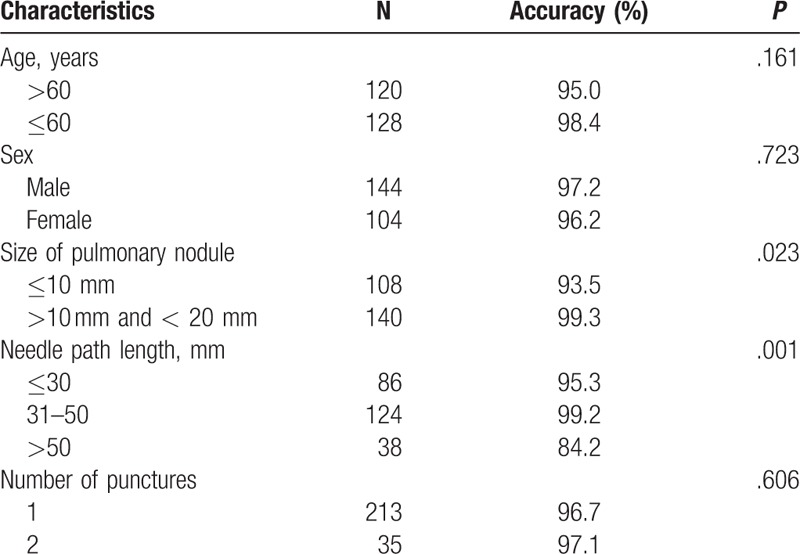
Diagnostic accuracy according to clinicopathological characteristics.

### 3.3 Complications

Pneumothorax as the most frequent complication of these procedures occurred in 40/248 (16.1%), all of the cases were small amount (<20%) and improved automatically without chest tube insertion. Pulmonary hemorrhage was found in 17 patients, showed as patchy consolidation surrounding the needle path in 12 cases, all typically self-limited, and hemoptysis in 5 patients, with 3 cases showed bloody sputum who improved untreated and 2 cases of small quantity (one in 20 mL, and another 10 mL), the bleeding stopped when treated with thrombin. No fatal adverse reactions occurred. Further, higher rates of pneumothorax were encountered in patients who underwent repeated puncture (*P = *.013) and where a long needle path length in the pulmonary parenchyma was used (*P = *.019, Table 3).

**Table 3:**
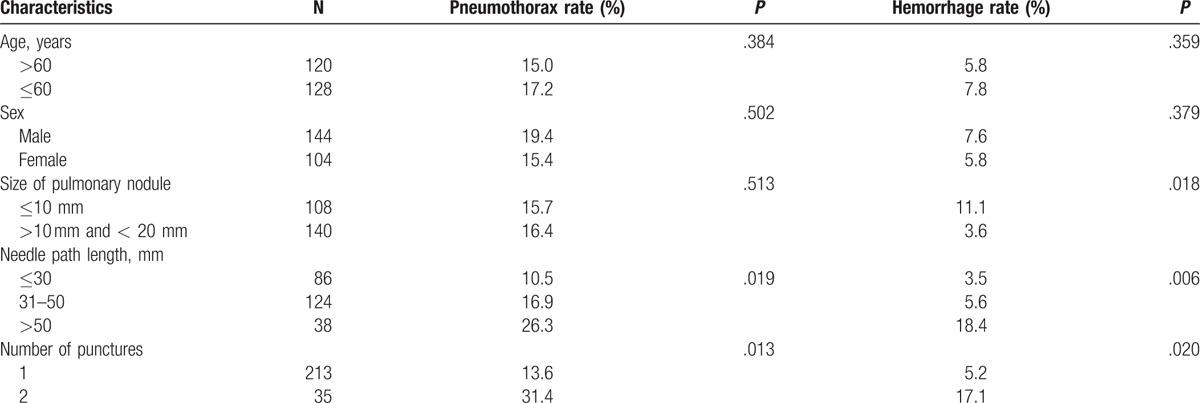
Pneumothorax and hemorrhage rates according to clinicopathological characteristics.

## 4 Discussion

The identification of SPN becomes more common because of the increased use of radiological technology. Therefore, it is important that clinicians become familiar with managing these nodules. The prevalence of malignant SPN ranged 20% to 40%, furthermore, the likelihood of malignancy increased in the older, which increased more than 1.2 times for every 10-year increase in age for patients aged between 55 to 75 years.^[8]^ It is critical to obtain histopathologic specimens for the diagnosis and treatment of SPN. Haaga reported the first case of percutaneous transthoracic CT guided biopsies, since then the technology developed constantly. The diagnostic yield with this procedure exceeds 75% to 81% in pulmonary lesion in the literature.^[9,10]^ As the minimally invasive nature, percutaneous pulmonary biopsy has become the procedure of choice for diagnosis of SPN because of high positive rate, slight injury and low charging. Fine needle aspiration biopsy was used in the early stages of percutaneous pulmonary biopsy, provides a cytological sample of exfoliated cells. As developed, more clinicians prefer core biopsy as it provides more tissue sample and permits more laboratory testing, enhancing diagnostic specificity significantly compared with cytology alone.^[11]^ Sputum cytology, bronchoscopy, and thoracoscope are commonly used in SPN diagnosis besides percutaneous lung biopsy.^[12,13]^ Sputum cytology is a convenient and noninvasive method, but the positive rate is extremely low. Bronchoscopy has a superior overall diagnostic yield of central lesions; nevertheless diagnosis value of SPN is limited. Thoracoscope is also not the ideal routine diagnostic methods for the prerequisite of general anesthesia, high risk of trauma and expense.

The mean diameter was 15 ± 4.8 mm in the group of 248 SPNs. An adequate sample for diagnosis was obtained in all of the 248 patients. The yield ratio of puncture was 100%, and the accuracy was 96.8%, consistent with the literature report.^[7]^ Smaller size of lung nodule presented a high technical difficulty and was associated with higher diagnostic failure rate. The diagnostic accuracy of large nodules group and small nodules group was 99.3% and 93.5%, respectively, indicating that diagnostic accuracy was significantly affected by the lesion size. For small nodule in diameter 10 mm or less, the imaging features should be carefully assessed before puncture to determine the appropriate needle path and draw points. Moreover, preoperative training should be strengthened for the poor tolerance patients, to improve the compliance and accuracy.

Previous study reported that the incidence of pneumothorax and hemorrhage was 8% to 64% and 26% to 33% in terms of complications.^[14]^ Pneumothorax as the most frequent complication of our study occurred in 40/248 (16.1%), all of the cases were small amount, and pulmonary hemorrhage was found in 17 patients. No fatal adverse reactions occurred. Accordingly, in previous series of CT-guided biopsy in the literature, pleural lesions were associated with a very low incidence of pneumothorax; as soon as the aerated lung was traversed, the risk rose considerably. In our study, higher rates of pneumothorax were encountered in patients who underwent repeated puncture and where a longer needle path length in the pulmonary parenchyma was used. The incidence of hemorrhage increased in larger lesions and longer needle path length. Lung biopsy under CT guidance does carry more risks including bleeding and pneumothorax than bronchoscopy. Forty cases of pneumothorax and 17 cases of bleeding occurred in the study, showed mild symptom, with no sever and life-threatening complication.

Choosing the appropriate diagnosis method could maximize the diagnosis accuracy and avoid the occurrence of adverse reactions according to the clinical features of SPN patients. The incidence of pneumothorax was greatly increased in condition that the nodules were close to the hilar or far from the surface, or with poor pulmonary function, adding pulmonary bullae adjacent with the needle path. Substituted diagnostic methods should be selected when the nodules are located in the vicinity of large vessels, which increasing the bleeding risk.

Several limitations of our study warrant discussion. First, this is a retrospective review, and therefore the selection bias does exist. Second, we performed this study at a single center with a relatively small sample size. Furthermore, the study lacks a control group. Further perspective controlled trial should be performed.

In conclusion, CT-PTNB has several advantages in diagnosing SPN. With skilled operation, strict-selected patients and precise location of the puncture, the diagnostic accuracy of CT-PTNB would be improved, and the complications reduced.

## Author contributions

**Conceptualization:** C. Xu.

**Data curation:** Y. Wang.

**Formal analysis:** C. Chi.

**Investigation:** Q. Zhang.

**Resources:** W. Wang, L. Yu.

**Software:** P. Zhan.

**Supervision:** Y. Lin.

**Writing – original draft:** Q. Yuan.
